# Environmental and spatial risk factors for the larval habitats of *Plasmodium knowlesi* vectors in Sabah, Malaysian Borneo

**DOI:** 10.1038/s41598-021-90893-1

**Published:** 2021-06-03

**Authors:** Isabel Byrne, Wilfredo Aure, Benny O. Manin, Indra Vythilingam, Heather M. Ferguson, Chris J. Drakeley, Tock H. Chua, Kimberly M. Fornace

**Affiliations:** 1 Faculty of Infectious and Tropical Diseases, London School of Hygiene and Tropical Medicine, Keppel Street, Bloomsbury, London, WCIE 7HT UK; 2 Faculty of Medicine and Health Sciences, Universiti Malaysia Sabah, Kota Kinabalu, Malaysia; 3 Research Institute for Tropical Medicine, Manila, Philippines; 4 Department of Parasitology, Faculty of Medicine, Universiti Malaya, Kuala Lumpur, Malaysia; 5 Institute of Biodiversity, Animal Health and Comparative Medicine, University of Glasgow, Glasgow, UK

**Keywords:** Malaria, Ecological epidemiology

## Abstract

Land-use changes, such as deforestation and agriculture, can influence mosquito vector populations and malaria transmission. These land-use changes have been linked to increased incidence in human cases of the zoonotic malaria *Plasmodium knowlesi* in Sabah, Malaysian Borneo. This study investigates whether these associations are partially driven by fine-scale land-use changes creating more favourable aquatic breeding habitats for *P. knowlesi* anopheline vectors. Using aerial remote sensing data, we developed a sampling frame representative of all land use types within a major focus of *P. knowlesi* transmission. From 2015 to 2016 monthly longitudinal surveys of larval habitats were collected in randomly selected areas stratified by land use type. Additional remote sensing data on environmental variables, land cover and landscape configuration were assembled for the study site. Risk factor analyses were performed over multiple spatial scales to determine associations between environmental and spatial variables and anopheline larval presence. Habitat fragmentation (300 m), aspect (350 m), distance to rubber plantations (100 m) and *Culex* larval presence were identified as risk factors for *Anopheles* breeding. Additionally, models were fit to determine the presence of potential larval habitats within the areas surveyed and used to generate a time-series of monthly predictive maps. These results indicate that land-use change and topography influence the suitability of larval habitats, and may partially explain the link between *P. knowlesi* incidence and deforestation. The predictive maps, and identification of the spatial scales at which risk factors are most influential may aid spatio-temporally targeted vector control interventions.

## Introduction

Malaysia has reached malaria pre-elimination status and is currently under review for certification malaria elimination in 2021. Despite strong progress in reducing the incidence of human malarias, disease control efforts have been hampered by the emergence and increase in human cases of the zoonotic *Plasmodium knowlesi*^1^*.* Previously misidentified as *P. malariae*, *P. knowlesi* was first identified as a public health threat in 2004^2^. Since then, the reported incidence of human cases of the simian malaria has increased, and *P. knowlesi* is now the most common cause of malaria in humans in Malaysia^3^. Most human cases of *P. knowlesi* occur in the Malaysian Bornean state of Sabah; with a large cluster in the northern district of Kudat, where *P. knowlesi* constituted 98% of malaria admissions in 2017^4,5^. Taking improvements in molecular diagnostics and surveillance into account, the increase in *P. knowlesi* cases in Malaysian Borneo likely represents a genuine incidence rise. Sabah is a global hotspot for deforestation, and a clear association between the rise of *P. knowlesi* incidence in humans and deforestation have been shown in Sabah^6,7^. While the mechanisms which underly this association are unknown, it has been suggested that they may be influenced by changes in macaque behaviour and densities, human behaviour and vector bionomics^8^.

*P. knowlesi* transmission is sustained by primary reservoir hosts: the long tailed, and pig tailed macaques (*Macaca fasicularis* and *Macaca nemestrina*). Spillover events occur when infected anopheline mosquito vectors feed on humans. Mosquitoes, including the *Anopheles* genus, have 4 main life stages. The juvenile egg, larva and pupa stages are aquatic, and the adult stage is terrestrial^9^. The primary vector of *P. knowlesi* in Sabah is *Anopheles balabacensis*; a member of the Leucosphyrus group of *Anopheles*^10^. *Anopheles balabacensis* are described as a primarily forest-dwelling species, with the larval stages preferring humid, shaded aquatic habitats^11^. The high levels of land-use change in Sabah are impacting this vector’s ecology. Deforestation separates habitats into fragments, bringing distinct ecosystems into closer contact, and can create new habitats at the forest fringe^12^. Higher abundances of *An. balabacensis* have recently been reported in disturbed, logged forest than in unmodified primary forest^13^. In Kudat, high densities have been found in shrub and bush habitats, often close to human settlements, deforested areas, plantations and farms^14^, and higher densities have been found in peri-domestic settings than in plantation and secondary forested sites^15^. A recent study of the ecology of *P. knowlesi* vectors over a wider geographic area in Sabah found different patterns of vector-habitat associations, with higher *An. balabacensis* abundances in farms and forest patches than peri-domestic settings, highlighting the local context of the findings from Kudat^16^. There are a number of suggested mechanisms which may explain these changes in *P. knowlesi* vector abundance and distribution in response to land use change in Sabah. Deforestation can change microclimate, vegetation and soil composition, possibly creating new habitat types for mosquito populations^6,17^. Brant et al*.*^13^ suggested that the changes in *P. knowlesi* vector abundance and distribution may be explained by an increased availability of larval habitats resulting from land-use change. Data on the fine-scale landscape factors which may mediate such a process, are largely unreported. However, the current knowledge on *An. balabacensis* vector ecology relies strongly on studies based on adult populations.

Kudat has seen extensive land-use change, with large areas of forest converted to palm oil and rubber plantations^18^. Rohani et al.^19^ characterised the breeding sites of *An. balabacensis* in Kudat, describing associations between water body types and the vector’s larvae. They reported *An. balabacensis* larvae in ground pools, tyre tracks, slow-flowing streams and swamps. The survey employed a ‘purposeful’ sampling design, sampling in locations where one would expect to find higher *An. balabacensis* densities. Study sites were selected based on high village incidence of *P. knowlesi* malaria and large vector populations. Their results, therefore, may not be representative of Kudat’s various habitat types and their different ecologies. The study did not describe the associations between breeding sites and their surrounding environment. Ageep et al*.*^20^ demonstrated the benefits of using geospatial tools (remote sensing and geographic information systems) to plan and execute spatially representative and randomly sampled larval surveys. The advantage of this study design is that one can acquire a dataset with a reasonable representation of all habitat types. This will result in an accurate representation of the variability of vector breeding sites across a study site, rather than an overrepresentation of habitats which are easy to access or for which there is a prior knowledge of association with larval presence^21^. Such data can provide a comprehensive picture of the whole vector population across the full range of land types in the study site. This could highlight areas where aquatic habitats likely or unlikely to be present, which may be useful for formulation of vector control strategies.

These spatially representative data additionally provide opportunities to examine how landscape configuration influences vector breeding sites^20^. Organisms interact with their surrounding environment at varying spatial extents, or “spatial scales”. These are the different distances over which environmental factors influence and determine the distribution of predators, food sources and breeding sites. As a result, the spatial distribution of aquatic habitats selected by female mosquitoes for oviposition is likely determined by interactions between mosquitoes and their environment occurring over varying spatial scales, rather than solely at the point of oviposition^22,23^.

We hypothesised that land-use change is increasing the availability of *Anopheles* vector larval habitats, resulting in an increase in human *P. knowlesi* incidence in Sabah. The aim of our study was to assess the associations between fine-scale landscape factors over multiple spatial scales and the presence of potential *P. knowlesi* vector larval habitats in an anthropogenically disturbed landscape of Kudat. The key objectives were to develop a larval survey sampling frame which was representative of the study site using aerial drone imagery, to use remote sensing (satellite and drone) data to assemble environmental and spatial covariates for the study site, and to identify the fine-scale landscape risk factors for *P. knowlesi* vector larval habitats at their most influential spatial scales. We also assessed key indicators for the presence of potential *P. knowlesi* larval habitats and predicted their presence within the study site over time. Together, this study illustrates the role of fine-scale land use to anopheline larval ecology and highlights potential targets for surveillance and control.

## Results

**Figure 1:**
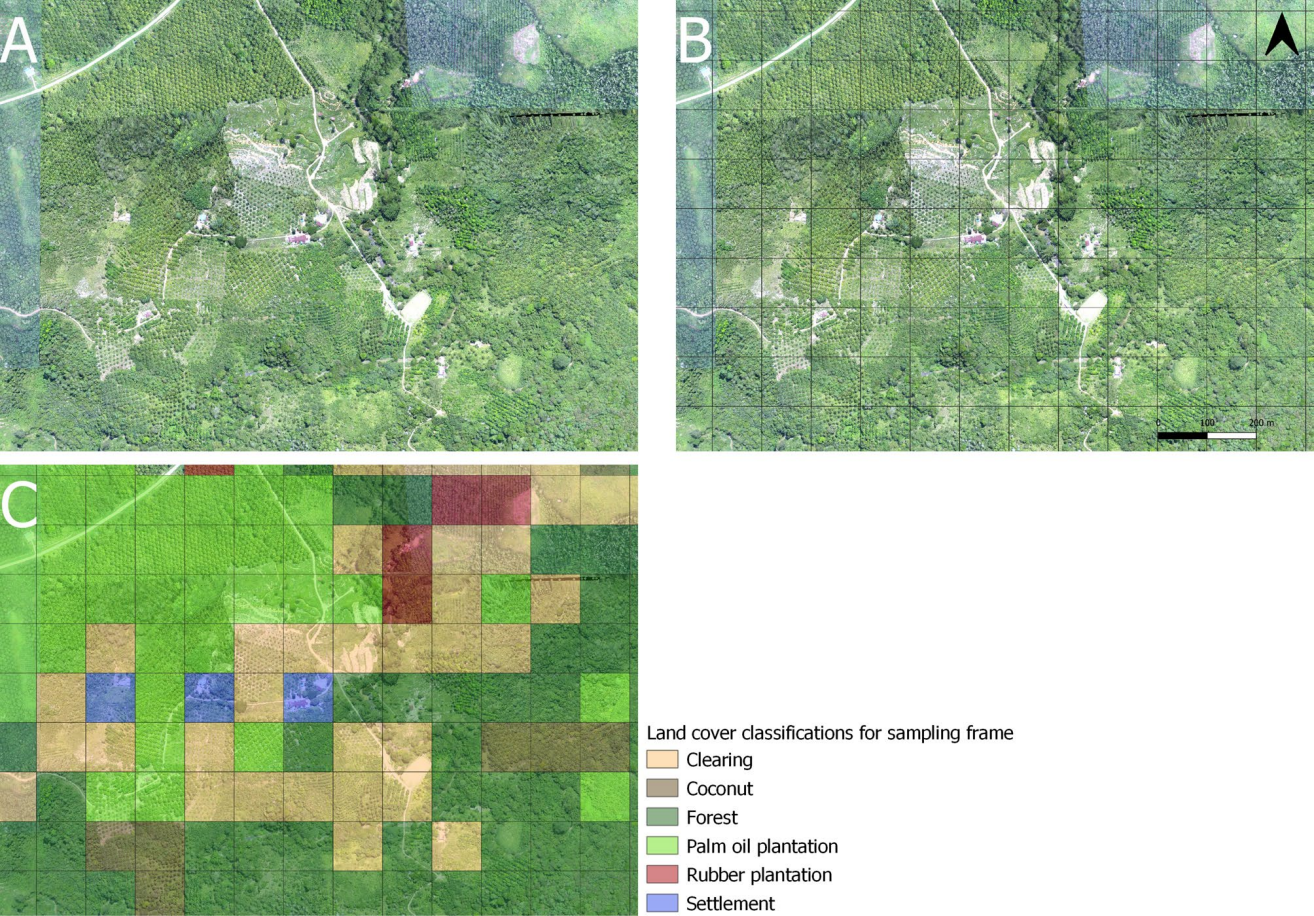
(**A**) Drone image of a sub-section of the sampling site. (**B**) The sub-section split into 100 × 100 m sampling blocks. (**C**) Sampling blocks classified by their predominant land use, used to create the larval survey sampling frame which ensured an even representation of habitats. All drone images collected by^24^.

This study area was highly fragmented and consisted of secondary forest, village areas, plantations and open areas. Prior to the start of larval collections, detailed high-resolution aerial imagery was generated through aerial drone surveys, using the methods described by Fornace et al.^24^. To define a sampling frame representative of all land cover types, we divided this study area into a 3 × 2 km grid of 600 sampling blocks of 100 × 100 m. We assigned each sampling block to a habitat strata based on visual identification of the predominant land type within the sampling block (Fig. 1). This yielded 217 sampling blocks classified as forest, 175 as clearing, 91 as palm oil plantation, 41 as rubber plantation, 37 as settlement and 39 as coconut plantation.

From the 600 blocks, we sampled the same 1 fixed block every month to capture temporal variation at the same sites. We additionally sampled at least 3 randomly selected blocks per land strata every month. This resulted in a total of 516 blocks which were sampled at least once throughout the sampling period (Table 1). 365 water bodies were sampled in total (Table 2). *Anopheles* larvae were collected from 95 water bodies, including artificial containers, borrow pits, ditches, irrigation canals, intermittent streams, leaf axils, ponds, puddles, rock pools, rivers, streams and tree holes. Of these *Anopheles* positive water bodies, Culex larvae were collected from 30 (32%), and Aedes larvae were collected from 3 (3%). A total of 25 *Anopheles* larvae were speciated further than the genus level, with 19 of these being identified as the primary *P. knowlesi* vector in Sabah *An. balabacencis* (75%).

**Table 1:** Numbers of sampling blocks sampled for water bodies and proportions of these which were positive for Anopheles and *An. balabacensis* larvae by habitat strata.

Sampling blocks		Number of sampling blocks sampled	Number of sampling blocks positive for water bodies	Number of sampling blocks positive for *Anopheles* larvae	Number of sampling blocks with *Anopheles* larvae speciated as * An. balabacensis*
Total		516	365 (0.71)	76 (0.21)	19 (0.2)
Water bodies per habitat strata	Clearing	84	58 (0.69)	14 (0.24)	1 (0.07)
	Coconut plantation	85	66 (0.78)	18 (0.27)	2 (0.1)
	Forest	82	62 (0.76)	9 (0.15)	3 (0.33)
	Palm oil plantation	91	65 (0.71)	13 (0.2)	2 (0.17)
	Rubber plantation	73	46 (0.63	12 (0.26)	7 (0.58)
	Settlement	101	68 (0.67)	10 (0.15)	4 (0.4)

Proportions included in brackets. Repeated visits to sampling blocks are included in this table.

**Table 2:** Numbers of water bodies sampled and proportions positive for Anopheles larvae by sampling block strata.

Water bodies		Number of water bodies sampled	Number of water bodies positive for *Anopheles* larvae	Number of water bodies with *Anopheles* larvae speciated as * An. balabacensis*
Total		365	95 (0.26)	19 (0.2)
Habitat strata	Clearing	58	15 (0.26)	1 (0.06)
	Coconut plantation	66	20 (0.30)	2 (0.1)
	Forest	62	12 (0.2)	3 (0.25)
	Palm oil plantation	65	15 (0.23)	2 (0.13)
	Rubber plantation	46	19 (0.41)	7 (0.37)
	Settlement	68	14 (0.21)	4 (0.29)

Proportions included in brackets. Repeated visits to sampling blocks are included in this table.

The number of Anopheles positive water bodies found per month ranged from 1 to 43 with a mean of 15 (± 11). The highest numbers of water bodies positive for Anopheles larvae were found in November and December, and the highest mean EVI and rainfall levels were recorded in February (Supplementary Information Fig. 3).

**Figure 2:**
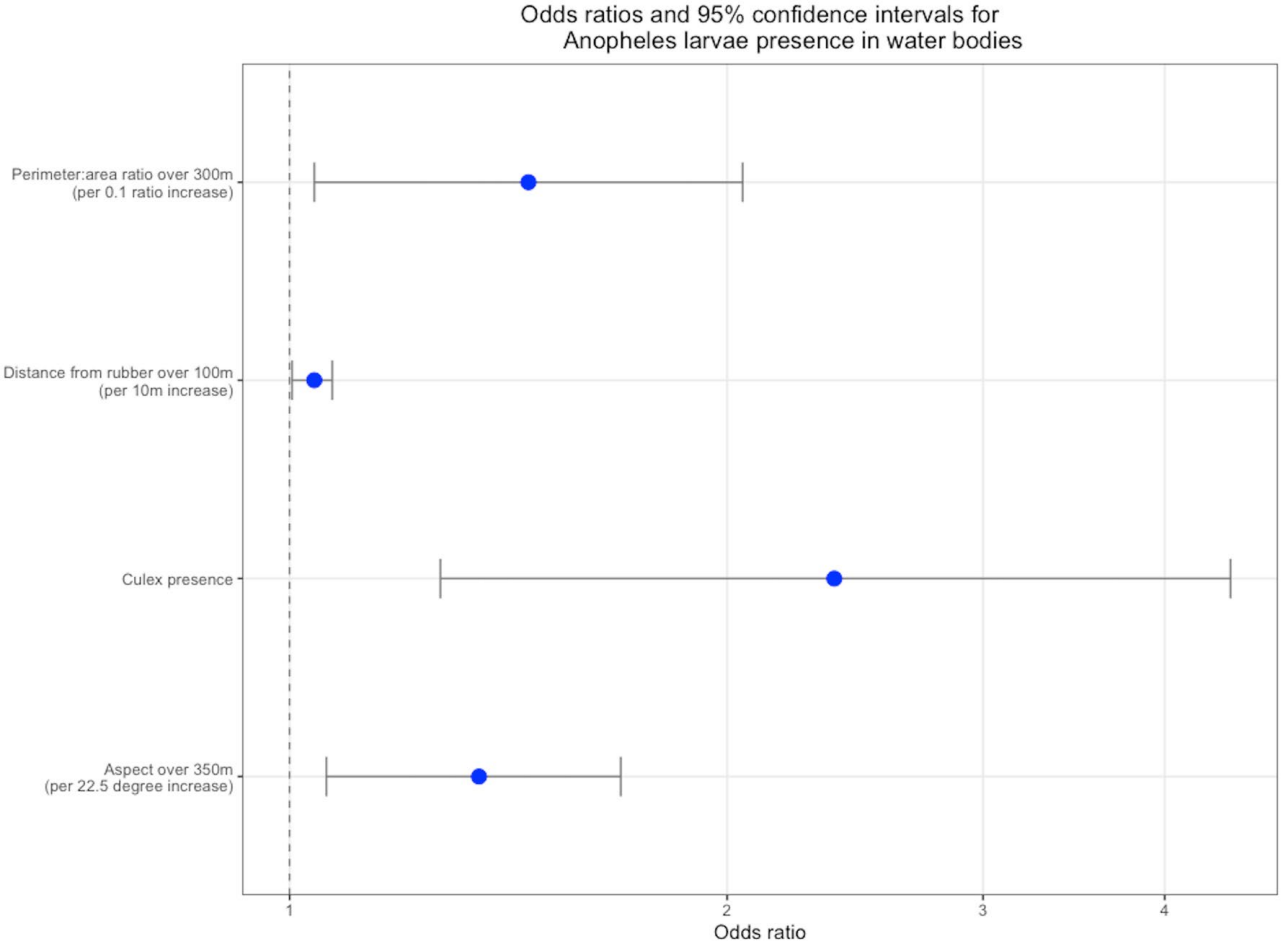
Odds ratios and 95% confidence intervals for risk factors for Anopheles larval habitats, at their most influential spatial scales.

To determine the effect of fine-scale landscape factor and land use patterns, we extracted remote sensing derived variables on land cover, fragmentation, rainfall and topography (Table 3) at buffer distances from 50 to 500 m, at 50 m intervals, from each surveyed water body (Supplementary Information Fig. 1). We additionally extracted variables aggregated to the 100 m^2^ level, for all sampling blocks within the survey site. We first fit models to determine the presence or absence of anopheline larvae in water bodies, (univariate model results presented in the Supplementary Information Table 1). Of the 185 univariate models run for the presence of *Anopheles* in the larval survey water bodies, 35 were significant at p < 0.2. When the significant variables were assessed for their most influential spatial scales, 12 variables were assessed for inclusion in the multivariate model. These variables comprised enhanced vegetation index (EVI) at 500 m, aspect at 350 m, distance from bush forest at 400 m, distance from rice agriculture at 250 m, distance from rice plantations over 100 m, perimeter: area ratio at 300 m, water body situated in a palm oil plantation, water body situated in an area of recent deforestation, shrub vegetation, dense vegetation, *Culex* mosquito larvae present in the water body and *Aedes* mosquito present in the water body. In the final multivariate model increases in mean perimeter: area ratio at a 300 m spatial scale, average distance from rubber plantations at a 100 m spatial scale, mean aspect at a 350 m spatial scale and *Culex* larvae presence were all positively associated with the presence of *Anopheles* larvae in aquatic habitats (Fig. 2). There was no association between the land class at the site of the water body and larval presence.

**Table 3:** Environmental and spatial covariates assessed and their sources.

Covariate	Source
Rainfall	NASA Tropical Rainfall Monitoring Mission (TRMM)^25^
Enhanced Vegetation Index (EVI) and Normalised Differential Vegetation Index (NDVI)	NASA Terra Moderate Resolution Imaging Spectroradiometer (MODIS)^26,27^
Elevation	NASA Terra ASTER global digital elevation model (DEM)^28^
Slope, aspect, topographic wetness index (TWI)	Derived from elevation raster
Land class water body situated in	Classified land cover map of Sabah, prepared as described by^29^
Distance of water body to 9 land classes: bush forest, rubber, coconut/mixed plantation, palm oil plantation, rice, built, grassland/ cleared land, intact forest, water	Classified land cover map of Sabah, prepared as described by^29^
Recent deforestation	Time series of drone imagery of the study site collected in 2014, prepared as described by^30^
Vegetation density and diversity	Aerial drone imagery of the study site
General habitat fragmentation	Extracted from classified land cover map

The methods used to extract each covariate from their corresponding raster are described in Supplementary Information Table 4.

**Figure 3:**
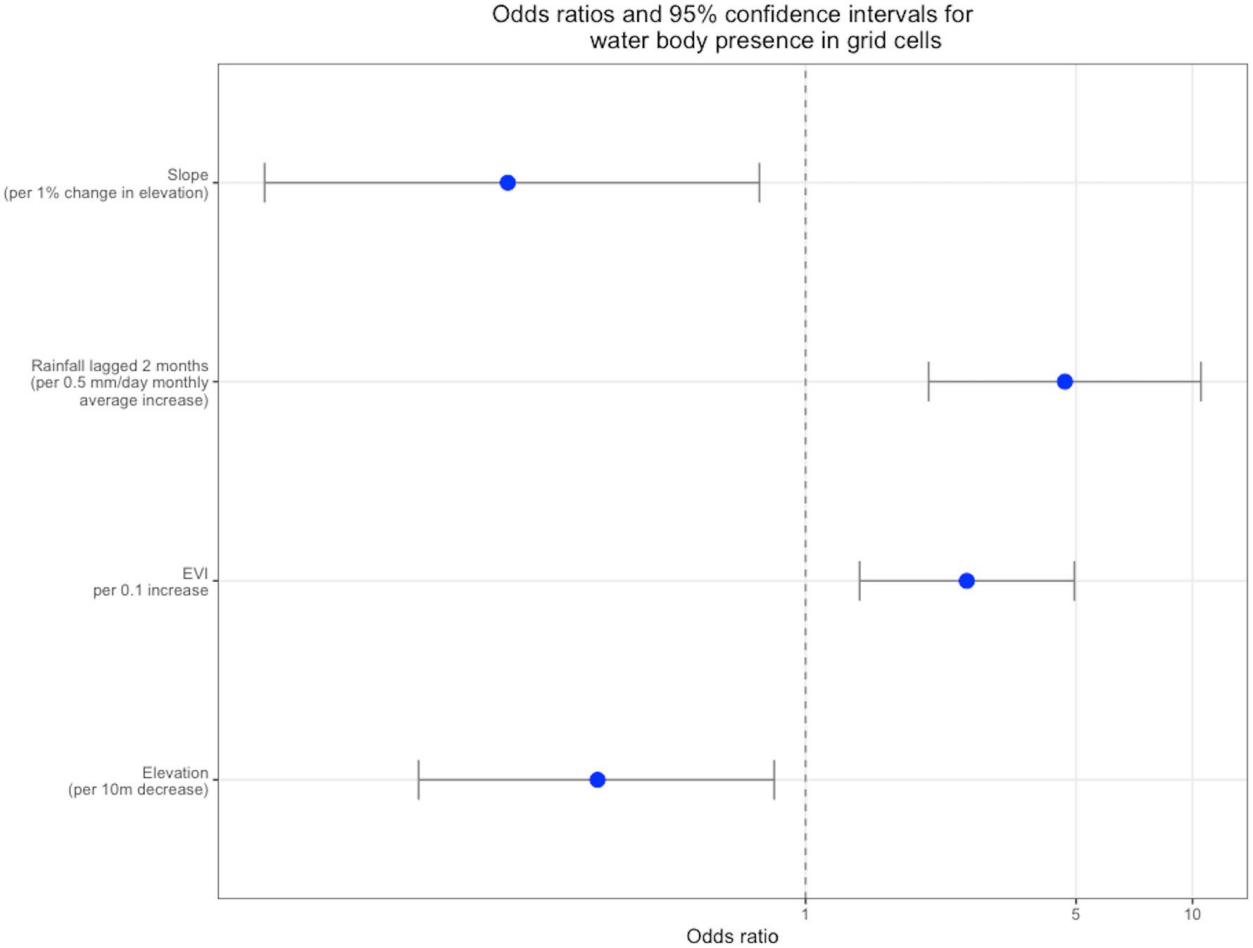
Odds ratios and 95% confidence intervals for risk factors for presence of water bodies within sampling blocks.

The second part of the analysis involved fitting two models to determine the presence or absence of water bodies within sampling blocks, and the presence or absence of anopheline larvae in sampling blocks. The univariate results are presented in the Supplementary Information Tables 2 and 3. Results of the final multivariate regression indicated that the presence of water bodies in sampling bodies was negatively associated with elevation and slope and positively with rainfall lagged by 2 months and EVI (Fig. 3). The Moran’s I statistic for residual spatial autocorrelation in the water body presence model was low, but significant (Moran’s I 0.16, 0.01). There was no residual temporal autocorrelation. Predictive power of the model as Area Under the Curve (AUC) was moderately strong (AUC 0.76, 95% CI 0.72–0.81). The four variables which comprised the final model for water body presence in sampling blocks were used to create a time-series of predictive maps over the study time period (Video 1). Although aspect and elevation were both significantly associated with *Anopheles* presence in sampling blocks in the univariate analysis (Supplementary Information Table 3), these variables did not remain significant in the multivariate analysis. Thus, none of the environmental factors investigated were significantly associated with presence of *Anopheles* larvae in sampling blocks. The Moran’s I statistics for residual spatial autocorrelation in the sampling block model for *Anopheles* larvae presence was not significant (Moran’s I 0.03, 0.06). There was no residual temporal autocorrelation, and the predictive power was moderately strong (AUC 0.75, 95% CI 0.71–0.80). In the final multivariate models for both water body presence and larval presence within sampling blocks, there were no associations between the majority land class of the sampling block and presence of the response variable.

In these initial models of water body presence in sampling blocks, collection month was retained as a random effect to account for correlations between repeat samples. In a second step, we investigated whether month of collection was the main source of variation in water body and *Anopheles* larvae presence by removing this random effect and examining the impact of the AUC. The predictive power for the water body models remained stable when month was removed as a random effect, with only a minor reduction in AUC from 0.761 to 0.756 (95% CI 0.71–0.80). This confirms temporal variation between months was not a major source of variation for water body presence. In contrast, the predictive power of the *Anopheles* model fell more substantially (0.75 to 0.60, 95% CI 0.53–0.67) when month was removed, indicating that temporal variation may be a more important determinant of larval presence.

## Discussion

The key objective of this study was to address the gap in our understanding of the role of land use change and fine-scale environmental factors in driving increases in incidence of human *P. knowlesi* by increasing the availability of vector larval habitats. We found positive associations between *Anopheles* larval presence and distance from agriculture, forest fragmentation and topology, and key environmental indicators for potential vector breeding sites in the study site. The probability of detecting *Anopheles* larvae in aquatic habitats did not very between land classes, and there was no association between deforestation and *Anopheles* larvae presence. To our knowledge, this is the first study which has systematically assessed the environmental and spatial risk factors for *Anopheles* vector breeding ecology in Kudat. We demonstrated how aerial and satellite-based remote sensing data can be used to make a study more robust by informing a spatially representative sampling frame, and to evaluate risk factors for vector breeding over multiple spatial scales, a methodology highly relevant to other vector-borne diseases.

Overall, the findings show that there are interactions at play between *Anopheles* vector breeding, distance from plantation agriculture, forest fragmentation and topology. The associations with forest fragmentation and distance from rubber plantations indicate that *An. balabacensis* vector ecology may be more complex than its previous descriptions as a primarily forest dwelling group, or the vector has adapted to the new environment resulted from changing land use patterns. This is supported by the lack of association between *Anopheles* positive water bodies and forested land types, and the lowest proportions of *Anopheles* larvae positive water bodies being found in forested land classes. These findings support our hypothesis that land use change is creating more suitable habitats for *Anopheles* vector larvae and this may be contributing to the higher *P. knowlesi* incidence in humans in Kudat. Our findings are consistent with recent findings of increased adult *An. balabacensis* abundance in disturbed forests, plantations, farms and close to human settlements^13–15^. The findings also align with those of Fornace et al*.*^29^, that fragmentation, aspect and agriculture are associated with increased risks of *P. knowlesi* exposure, and may explain some of the mechanisms underlying this link, whereby these changes to land cover may increase potential for *P. knowlesi* vector proliferation and disease transmission.

*Culex* larvae were collected from roughly one third of the water bodies which were positive for *Anopheles* larvae, resulting in a strong positive association between these species’ larval presence. This may be driven by finer-scale micro habitat characteristics which were not analysed in this study, such as the physical and chemical characteristics of the water bodies which may create favourable conditions for oviposition in both species. However, further studies are needed to investigate this further.

The ecological processes which determine vector breeding ecology likely occur over multiple spatial scales^8,22,31–33^; however these scale-dependent effects are rarely considered in standard investigations of larval ecology. This study used a novel data-driven approach to incorporate and assess the contribution of environmental variables acting across different spatial scales (from 50 to 500 m around the larval collection points). The results showed that fragmentation, distance from agriculture and aspect were most strongly associated with larvae positive sites, each at different spatial scales. Habitat fragmentation, measured as perimeter: area ratio, over a 300 m buffer radius was positively associated with the presence of *Anopheles* breeding sites. Perimeter to area ratio is a strong and widely used indicator for habitat fragmentation^34^. Lower habitat patch size within a given area results in higher perimeter to area ratio, meaning that the area is constitutes smaller islands or “fragments” of habitat making up a complex mosaic, rather than larger homogenous patches of habitats^35^. Fragmented landscapes form a patchwork of land types and can expose more forest fringe, providing more potential larval habitats than one continuous patch^36^. This association may explain the findings by Fornace et al*.*^29^, that fragmented landscapes contribute to increased human exposure to *P. knowlesi* in Sabah. We additionally identified that mean proximity to rubber plantations was associated with increased risk of larval presence, and the highest proportions of *Anopheles* larvae were found in sampling blocks which were classified as rubber plantations. Although adult malaria vectors have been reported from rubber plantations in Thailand, Indonesia, and Malaysia^37–39^, there is limited evidence for anopheline breeding around rubber plantations in South East Asia. These habitats are, however, inhabited by *P. knowlesi* reservoir macaques^40^, and breeding in close proximity to macaque habitats may be beneficial to vectors as they can provide essential bloodmeals before oviposition. The rubber farmers in these habitats may also be a source of bloodmeals. The association of larval presence close to, but not within rubber plantations is consistent with previous reports of malaria vectors breeding on the outskirts of plantations in Thailand^41^. The central tendency of aspect over a 350 m radius was associated with *Anopheles* larvae presence. This finding supports, and may offer partial explanation to the findings of Brock et al*.*^8^, who found mean aspect over 1–2 km to be a potential determinant of household *P. knowlesi* infection risk in Northern Sabah. Aspect is highly correlated with agriculture type in Kudat and the association may arise from the availability of aquatic larval habitats in local agriculture^8^. While the mechanisms underlying these associations and the varying spatial scales over which they occur may be due to complex ecosystem processes, the results are useful in identifying the most influential spatial scales at which these fine-scale landscape risk factors work.

Additionally, we demonstrate how remote sensing data can be used to design sampling frames and stratify by habitat type. Although larval surveys are routinely conducted, these are typically only done in areas with water bodies. Quantifying the availability of aquatic habitats across different land types provides further insight into how land use change impacts larval distribution by identifying areas where no aquatic habitats are likely to be present. This sampling approach allowed us to identify *An. balabacensis* larvae in water bodies which are not usually surveyed for *Anopheles* larvae. The sampling block-level analysis provided key temporal indicators of water body presence within the study site. The results show that the presence of water bodies is largely governed by topographic and climactic variables; slope, elevation, rainfall levels two months prior and EVI. There were no strong indicators for the presence of *Anopheles* larvae within sampling-blocks. The experimental removal of collection month as a random effect in this model greatly reduced its predictive power, indicating that temporal trends may drive some of this variation. The lack of strong association with any of the environmental variables may also be due to the smaller sample size of larval presence compared to water body presence, or due to the more complex relationships between vector breeding, agriculture and fragmentation. Nevertheless, identification of factors associated with water bodies allows prioritisation of areas to sample or to target for preventative control programs and larval source management; for example, choosing areas to sample or treat based on topography and rainfall within the past two months.

While this study has generated several new insights into the larval ecology of *P. knowles*i vectors, it has several important limitations. As we aimed to characterise fine-scale environmental risk factors, with 500 m as the maximum spatial extent of prediction, this study cannot be used to generalise about larval distribution over wider spatial scales or predict over larger areas. As this study area was highly disturbed and in close proximity to human settlements, future studies could evaluate larval distribution within primary forests or across disturbance gradients. We were also limited by the water bodies which we could reach. It is possible that some potential larval habitats such as tree holes higher in the canopy and water collecting plants such as Bromeliads may have been missed in this sampling frame. High mortality rates when rearing *Anopheles* larvae, and the unavailability of molecular tools in the field to identify mosquitoes to the species level, meant that only a minority of *Anopheles* larvae could be speciated. Seventy six percent of the speciated *Anopheles* larvae were, however, identified as *An. balabacensis*, the primary *P. knowlesi* vector in the study region. We can therefore assume that a high majority of the *Anopheles* larvae from which the conclusions of this study are drawn, were of the *An. balabacensis* species. This indicates that the results presented in this study are directly relevant to *P. knowlesi* transmission. The study is also limited by the fact that it was conducted over one year and cannot be used to examine temporal fluctuations in vector breeding sites between years. More extensive long-term studies could additionally characterise breeding sites of different *Anopheles* species and confirm the majority within this area were *An. balabacensis.*

Despite these limitations, this study sheds important insights on anopheline vector ecology relevant for *P. knowlesi* transmission. Additionally, we develop a new methodology using drones to collect aerial imagery to define sampling frames which are representative of the land types present. Despite the complexities of land cover and landscape aspects associated with *P. knowlesi* breeding, it is clear from the results that *P. knowlesi* vectors are not strictly forest breeding in Kudat. Many of the results correspond with, and may offer partial explanation to the mechanisms underlying findings by Fornace et al*.*^1^ on the contribution of agriculture, topography and habitat configuration to human *P. knowlesi* exposure. We have provided key indicators which can inform the future surveys to be used in planning, and shown how datasets derived from freely available remote sensing sources and drone technology can be used to interrogate how fine-scale landscape factors are related to *P. knowlesi* vector breeding ecology.

## Methods

### Study site

**Figure 4:**
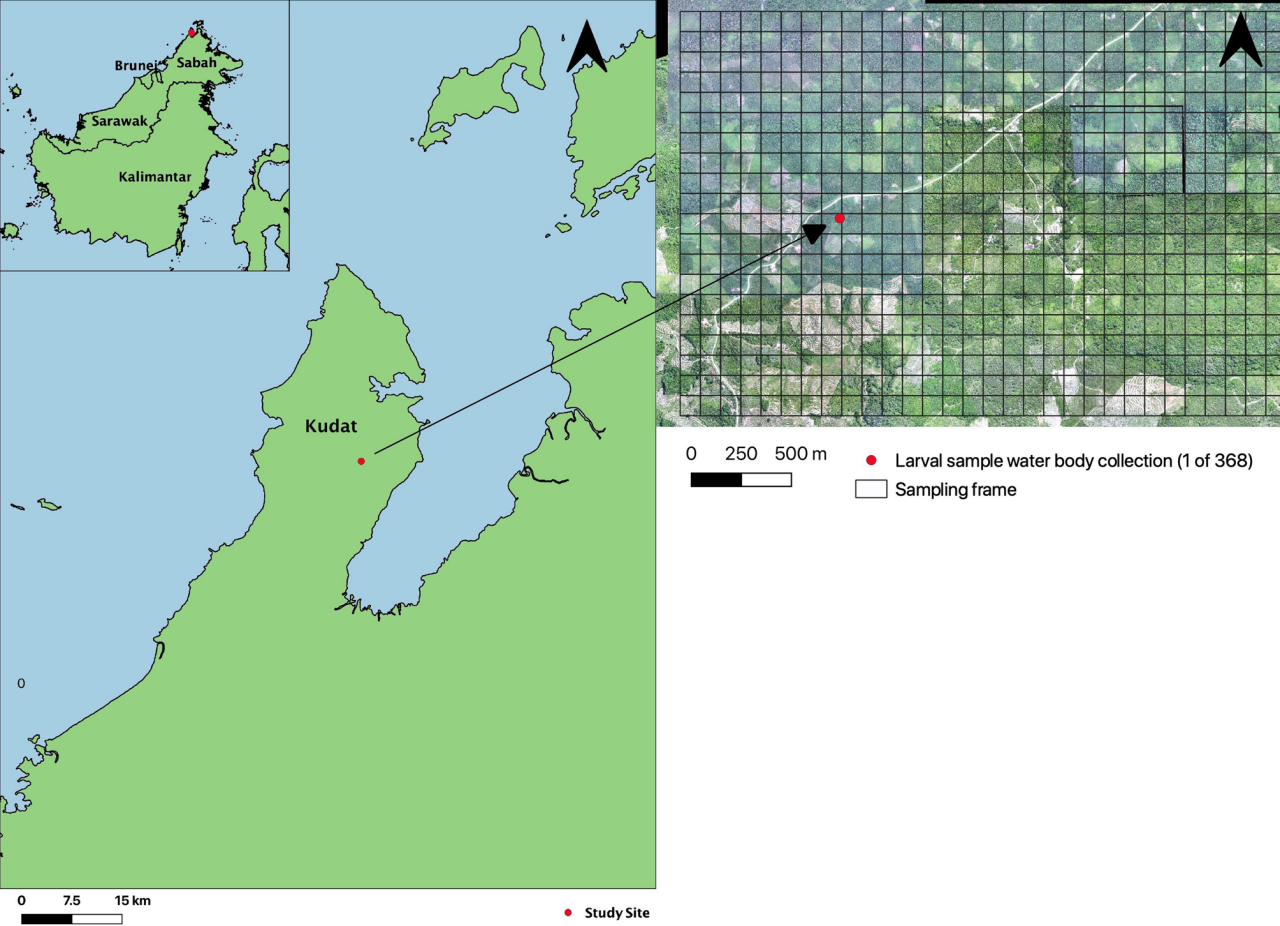
Map of Sabah, Malaysian Borneo, including drone image of sampling site in Kudat.

Kudat experiences a tropical climate, with temperatures averaging 32 °C in lowlands, and 21 °C in highlands (Fig. 4). Rainfall is frequent throughout the year, with higher volumes during the November to March north-east monsoons^14^. The landscape is comprised of lowland secondary forest, with extensive conversion to palm oil and rubber plantations^18^.

### Larval survey

A randomly stratified longitudinal larval survey was completed from May 11th 2015 to April 14th 2016 within a major transmission focus of *P. knowlesi* in Kudat, Sabah in Malaysian Borneo, previously described by Fornace et al.^1^. The survey was conducted within a 3 × 2 km grid composed of 600 sampling blocks of 100 × 100 m. To ensure an even representation of habitats sampled, drone imagery was used to classify each sampling block into 6 different land types based on visual classification of predominant vegetation by local field staff. The field staff were familiar with both the landscape and interpreting drone imagery using standardised guidelines describing each land type (Fig. 1). Every month, at least 3 random blocks from each land stratum were surveyed. Additionally, a single fixed block per strata was sampled every month to evaluate temporal trends. During the surveys every potential aquatic habitat which was within-reach of field technicians on the ground in the sampling block was sampled for mosquito larvae by larval dipping. Larvae were collected from water bodies by the conventional 10-dipping^42^. In smaller aquatic habits such as tree holes where dipping was not possible, a plastic pipette was used^43^. The GPS point of each sample site was recorded. Collected larvae were sorted by land use type and larval habitat and brought to the field laboratory for rearing to adult stages. Identification of adult mosquitoes was by microscope, and based on keys^44,45^.

### Remote sensing data analysis

**Figure 5:**
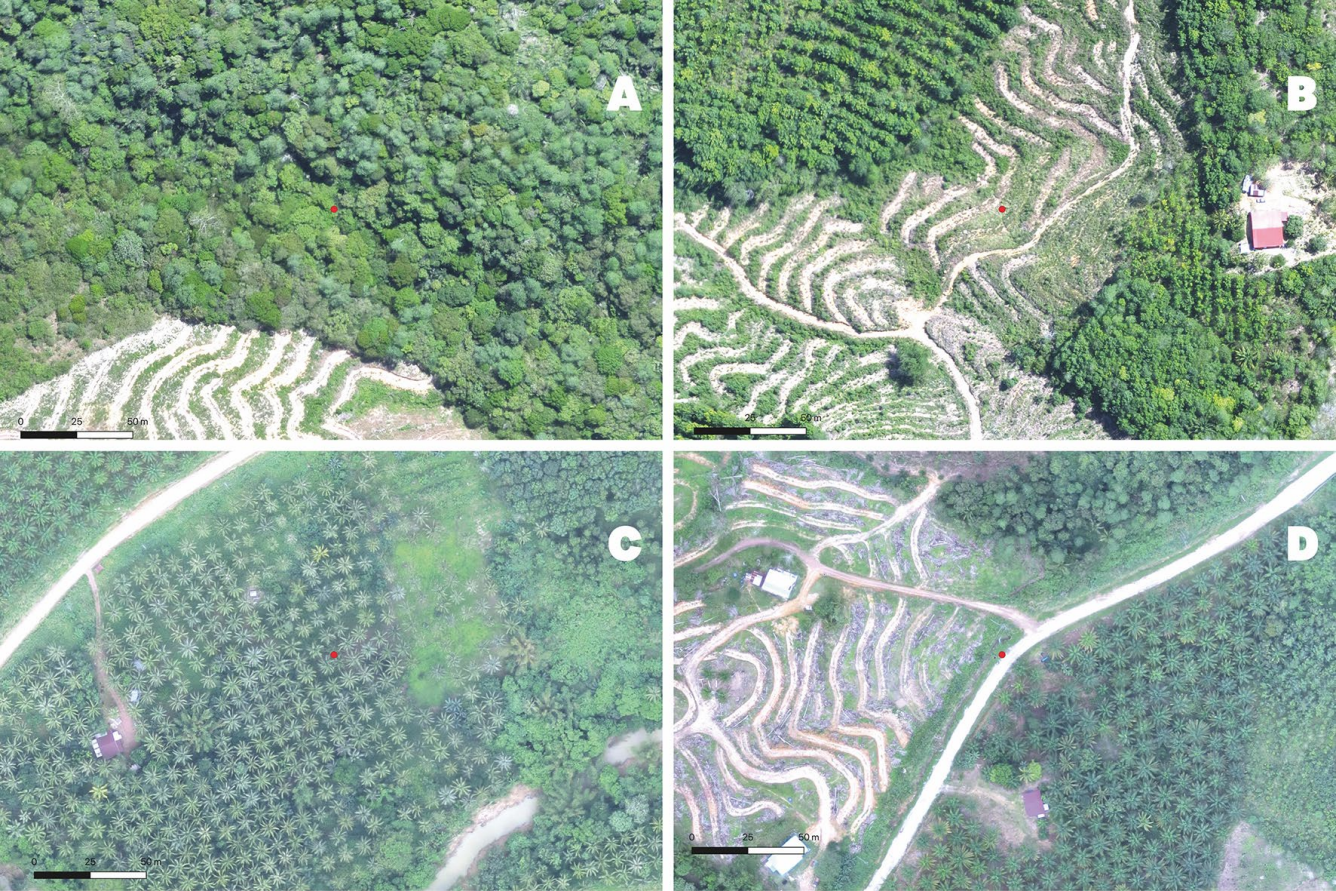
Examples of vegetation density levels. (**A**) Dense vegetation, (**B**) Patchy vegetation, (**C**) Planted vegetation, (**D**) Sparse vegetation. The red point represents the water body being categorised.

**Figure 6:**
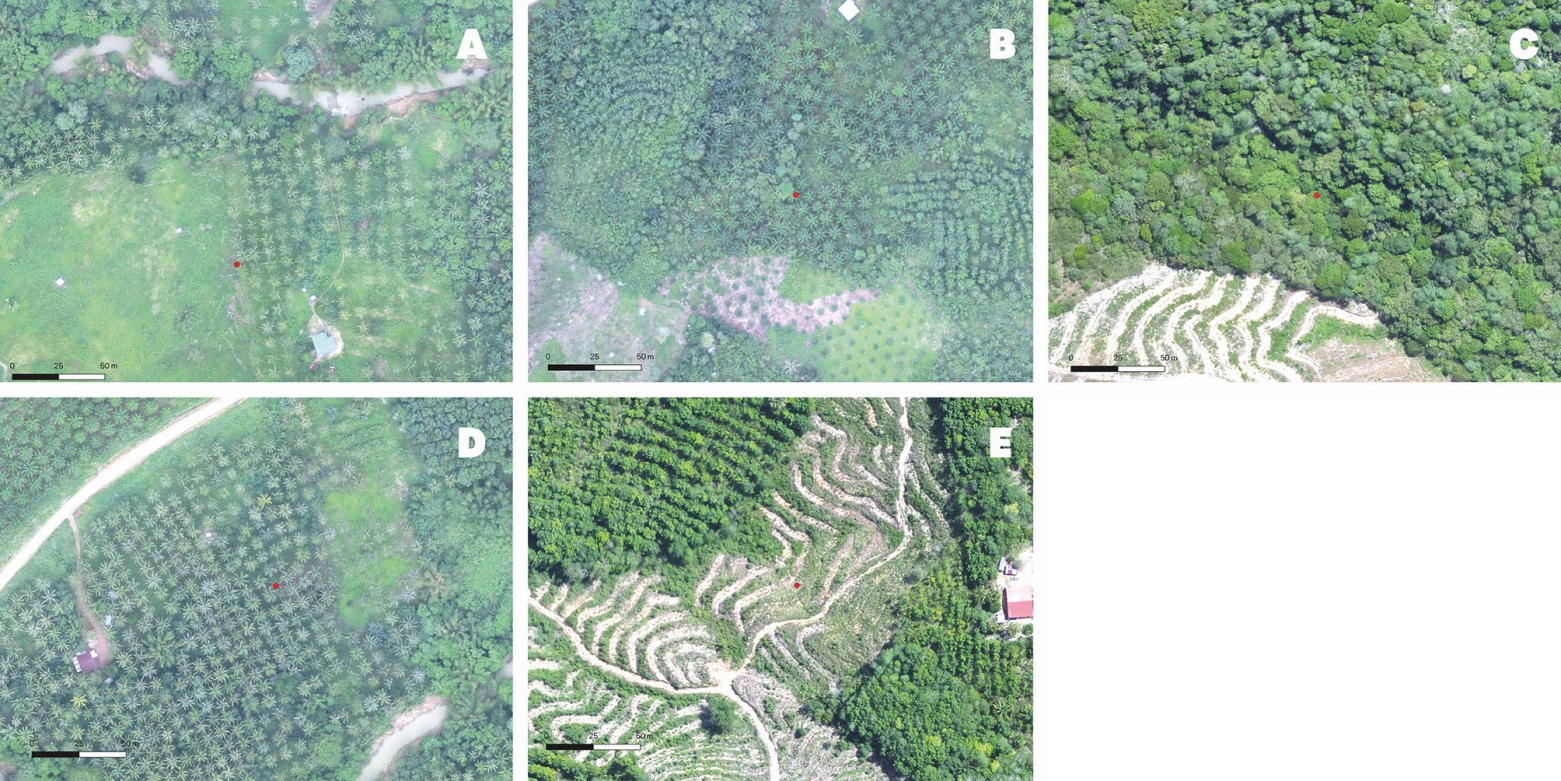
Example of vegetation diversity levels estimated from UAV imagery. (**A**) Edge, in this example monoculture and shrub, (**B**) Mixed-farmed, (**C**) Mixed forest, (**D**) Monoculture. The red point represents the water body being categorised.

We extracted the covariates to be assessed as risk factors from the drone and remote sensing data described in Table 3. Slope, aspect and topographic wetness index were calculated from the elevation raster in ArcGIS (10.8.1). The distance from different land cover classifications were calculated as Euclidean Distance in ArcGIS. EVI and NDVI were filtered for pixel quality, and raster values were scaled to a factor of 0.0001. Mean habitat fragmentation indices were calculated using the “landscapemetrics” package in R (v1.2.1335)^46^. The indices assessed were perimeter: area ratio (the ratio of habitat patch perimeter length to total patch area), shape index (measure of patch shape complexity adjusted for size of patch) and fractal dimension (degree of patch complexity across a number of spatial scales)^35,47^. Drone images of the sampling frame were visualised in QGIS (3.12) and the density and diversity of the vegetation surrounding each larval sampling site were qualitatively assessed. Vegetation density was categorised as dense (tightly-packed vegetation with no clear patches of forest floor), patchy (some low canopy with patches of low lying shrubbery and forest floor visible), planted (evenly spaced vegetation of the same species) and sparse (large portions of ground visible and little to no vegetation nearby) (Fig. 5). Vegetation diversity was categorised as edge (a visible transition between forest and another form of vegetation), mixed-farmed (mixture of planted species and natural growth), mixed forest (mixture of natural growth), monoculture (single species evenly planted) and shrub (low canopy level of majority of vegetation) (Fig. 6). Number of months since a deforestation event in 2014 was calculated for each sampling block using a series of 4 classified images of deforestation. Full details of the preparation of environmental and spatial covariates are explained in Supplementary Information Table 4, with visual examples of distance, EVI, rainfall and topographic rasters in Supplementary Information Fig. 1.

The mean and standard deviation of the covariates in Table 3, aside from time since deforestation and vegetation density and diversity, were extracted at 10 buffer radii (50–500 m in 50 m intervals), using the “raster” package in R. These buffer radii were used as a proxy for the spatial scales at which associations between covariates and *Anopheles* breeding may occur (Supplementary Information Fig. 2).

### Statistical analysis

All statistical models were built and run in R using the “lme4” package. Univariate binomial repeated measure mixed-effect logistic regression models were run for each covariate at each spatial scale (buffer radius). The primary outcome of this analysis was a binary presence or absence of larvae, and sampling block and collection month were included as random effects. All variables with p < 0.2 were assessed for inclusion in the multivariate analysis. Variables which were significant over multiple spatial scales or over multiple fragmentation indices were compared, and single variables were selected based on lower Akaike Information Criterion (AIC). The final multivariate model was developed using a foreword stepwise procedure, retaining all variables significant at p < 0.05 and assessing each step for reduction in AIC and interactions.

Two sampling block-level risk factor analyses were undertaken. The outcomes were the presence or absence of water bodies, and the presence or absence of *Anopheles* larvae, within sampling blocks. The variables assessed in the sampling block level analyses comprised the mean EVI, NDVI and monthly rainfall for collection month and lagged by 1 and 2 months, distance from a large water body, elevation, slope, aspect, TWI, the majority land class for each sampling block, and a binary variable for whether deforestation had occurred in each sampling block. These were analysed using the logistic regression procedure described above, with collection month as a random effect. To avoid overfitting these models by including sampling block as both an outcome and a random effect, sampling block was not included as a random effect. Residual spatial autocorrelation for risk of water body or *Anopheles* larval presence in sampling blocks was assessed using Moran’s I. Residual temporal autocorrelation functions and partial autocorrelation functions were also assessed for significance. The predictive power of the sampling block models were assessed by AUC. To determine whether collection month was a key source of variation in the sampling block-level models, it was experimentally removed, and AUC was examined. The results of the multivariate model for water body presence were used to predict the presence and absence of water bodies in each sampling block cell over the study period.

### Ethics

The data analysis (Ref: 22082, 19/05/2020) and larval survey (Ref: 6302, 19/12/12) were approved by the London School of Hygiene and Tropical Medicine Research Ethics Committee. This project was approved by the NMRR Ministry of Health Malaysia (NMRR-12-786-13048).

## Supplementary Information

Supplementary Information 1.

Supplementary Video 1.
