# Supplementary material for: Environmental and spatial risk factors for the larval habitats of Plasmodium knowlesi vectors in Sabah, Malaysian Borneo
Source: Sci Rep. 2021 Jun 3;11:11810. doi: 10.1038/s41598-021-90893-1 (PMC8175559; doi:10.1038/s41598-021-90893-1)
Supplement: Supplementary file 1 — Supplementary Information 1. [file 41598_2021_90893_MOESM1_ESM.docx]

# Supplementary Information

Supplementary Information Table 1: Results of univariate logistic regression analysis for each covariate at each scale for presence of Anopheles larvae in water bodies. Covariates in bold font were significant in the bivariate analysis (p < 0.2). Covariates significant at a single spatial scale were selected for stepwise selection into the final multivariate logistic regression model. If p < 0.2 on multiple scales for the same covariate, the bivariate model with the lowest likelihood ratio score and AIC score was selected for inclusion in the final model.

| Variable | Scale | Odds Ratio | 95% CI | P-value |
| --- | --- | --- | --- | --- |
| TRMM | 250m | 1.18 | 0.75, 1.85 | 0.46 |
| TRMM lag | 250m | 1.05 | 0.71, 1.56 | 0.83 |
| EVI | **Point** | **0.65** | **0.47, 0.89** | **0.01** |
|  | **250m** | **0.76** | **0.56, 1.05** | **0.09** |
|  | **500m** | **0.73** | **0.54, 1.01** | **0.06** |
| NDVI | Point | 1.05 | 0.76, 1.46 | 0.77 |
|  | 250m | 1.10 | 0.79, 1.54 | 0.55 |
|  | 500m | 1.01 | 0.73, 1.4 | 0.96 |
| Elevation | Point | 1.00 | 0.76, 1.32 | 0.99 |
|  | 50m | 0.98 | 0.72, 1.32 | 0.88 |
|  | 100m | 1.01 | 0.74, 1.38 | 0.97 |
|  | 150m | 1.02 | 0.75, 1.39 | 0.88 |
|  | 200m | 1.03 | 0.75, 1.41 | 0.85 |
|  | 250m | 1.04 | 0.75, 1.42 | 0.83 |
|  | 300m | 1.03 | 0.75, 1.43 | 0.84 |
|  | 350m | 1.02 | 0.74, 1.42 | 0.89 |
|  | 400m | 1.01 | 0.73, 1.41 | 0.95 |
|  | 450m | 0.99 | 0.71, 1.39 | 0.95 |
|  | 500m | 0.97 | 0.68, 1.37 | 0.85 |
| Slope | Point | 0.89 | 0.68, 1.16 | 0.39 |
|  | 50m | 0.96 | 0.72, 1.29 | 0.79 |
|  | 100m | 0.95 | 0.7, 1.28 | 0.72 |
|  | 150m | 0.99 | 0.73, 1.34 | 0.92 |
|  | 200m | 1.02 | 0.745, 1.39 | 0.92 |
|  | 250m | 1.00 | 0.73, 1.39 | 0.98 |
|  | 300m | 1.00 | 0.72, 1.39 | 1.00 |
|  | 350m | 0.98 | 0.7, 1.36 | 0.89 |
|  | 400m | 0.94 | 0.67, 1.32 | 1.36 |
|  | 450m | 0.90 | 0.64, 1.28 | 0.56 |
|  | 500m | 0.89 | 0.62, 1.26 | 0.51 |
| Aspect | **Point** | **0.79** | **0.6, 1.05** | **0.10** |
|  | 50m | 0.89 | 0.67, 1.18 | 0.40 |
|  | 100m | 0.97 | 0.73, 1.29 | 0.84 |
|  | 150m | 1.05 | 0.79, 1.4 | 0.73 |
|  | **200m** | **1.26** | **0.95, 1.67** | **0.11** |
|  | **250m** | **1.37** | **1.03, 1.82** | **0.03** |
|  | **300m** | **1.43** | **1.07, 1.91** | **0.01** |
|  | **350m** | **1.41** | **1.04, 1.89** | **0.02** |
|  | **400m** | **1.36** | **1.02, 1.82** | **0.04** |
|  | 450m | 1.18 | 0.89, 1.58 | 0.25 |
|  | 500m | 1.23 | 0.92, 1.66 | 0.16 |
| TWI | Point | 1.07 | 0.81, 1.41 | 0.63 |
|  | 50m | 1.03 | 0.76, 1.39 | 0.86 |
|  | 100m | 1.04 | 0.76, 1.42 | 0.82 |
|  | 150m | 1.00 | 0.73, 1.37 | 0.99 |
|  | 200m | 0.97 | 0.7, 1.33 | 0.83 |
|  | 250m | 0.97 | 0.7, 1.35 | 0.87 |
|  | 300m | 0.97 | 0.69, 1.35 | 0.85 |
|  | 350m | 0.99 | 0.7, 1.39 | 0.94 |
|  | 400m | 1.02 | 0.72, 1.44 | 0.90 |
|  | 450m | 1.08 | 0.76, 1.53 | 0.68 |
|  | 500m | 1.11 | 0.78, 1.58 | 0.57 |
| Land class site situated in (point) | Bush Forest | 0.63 | 0.17, 2.31 | 0.49 |
|  | Rubber | 0.43 | 0.12, 1.59 | 0.21 |
|  | **Coconut/ Mixed Agri** | **0.35** | **0.09, 1.45** | **0.15** |
|  | **Palm Oil Plantation** | **0.29** | **0.06, 1.38** | **0.12** |
|  | **Built Environment** | **0.33** | **0.8, 1.42** | **0.14** |
|  | Clearing/ Grassland | 0.86 | 0.25, 3 | 0.81 |
| Distance to house | Point | 1.00 | 0.73, 1.36 | 0.98 |
|  | 50m | 0.97 | 0.69, 1.37 | 0.86 |
|  | 100m | 0.91 | 0.65, 1.29 | 0.60 |
|  | 150m | 0.98 | 0.7, 1.38 | 0.93 |
|  | 200m | 0.90 | 0.64, 1.27 | 0.55 |
|  | 250m | 0.92 | 0.65, 1.29 | 0.63 |
|  | 300m | 0.98 | 0.7, 1.37 | 0.91 |
|  | 350m | 1.01 | 0.72, 1.41 | 0.97 |
|  | 400m | 1.13 | 0.81, 1.59 | 0.47 |
|  | 450m | 1.09 | 0.82, 1.47 | 0.54 |
|  | 500m | 1.12 | 0.86, 1.45 | 0.39 |
| Distance to recent deforestation | **Point** | **0.81** | **0.59, 1.11** | **0.18** |
|  | 50m | 1.11 | 0.8, 1.55 | 0.54 |
|  | 100m | 0.95 | 0.68, 1.35 | 0.79 |
|  | 150m | 0.97 | 0.69, 1.36 | 0.86 |
|  | 200m | 0.94 | 0.68, 1.3 | 0.71 |
| Distance to built environment | Point | 0.85 | 0.61, 1.18 | 0.32 |
|  | 50m | 0.95 | 0.69, 1.31 | 0.76 |
|  | 100m | 1.01 | 0.73, 1.4 | 0.94 |
|  | 150m | 1.05 | 0.77, 1.44 | 0.74 |
|  | 200m | 0.87 | 0.62, 1.22 | 0.42 |
|  | 250m | 0.95 | 0.71, 1.29 | 0.76 |
|  | 300m | 1.05 | 0.79, 0.38 | 0.75 |
|  | 350m | 1.06 | 0.82, 1.38 | 0.63 |
|  | 400m | 1.01 | 0.77, 1.32 | 0.97 |
|  | 450m | 1.13 | 0.89, 1.44 | 0.33 |
| Distance to large water body | Point | 0.79 | 0.49, 1.24 | 0.30 |
|  | 50m | 0.87 | 0.61, 1.24 | 0.43 |
|  | 100m | 0.87 | 0.61, 1.24 | 0.43 |
|  | 150m | 0.87 | 0.61, 1.24 | 0.44 |
|  | 200m | 0.86 | 0.6, 1.23 | 0.41 |
|  | 250m | 0.87 | 0.6, 1.25 | 0.44 |
|  | 300m | 0.86 | 0.6, 1.24 | 0.42 |
|  | 350m | 0.87 | 0.61, 1.25 | 0.45 |
|  | 400m | 0.85 | 0.6, 1.21 | 0.36 |
|  | 450m | 0.83 | 0.58, 1.18 | 0.30 |
|  | 500m | 0.82 | 0.58, 1.16 | 0.26 |
| Distance to clearing | Point | 0.86 | 0.58, 1.26 | 0.43 |
|  | 50m | 1.08 | 0.8, 1.46 | 0.63 |
|  | 100m | 1.02 | 0.75, 1.39 | 0.90 |
|  | 150m | 0.91 | 0.65, 1.27 | 0.57 |
|  | 200m | 0.89 | 0.64, 0.13 | 0.50 |
|  | 250m | 0.95 | 0.69, 1.3 | 0.75 |
|  | 300m | 0.96 | 0.72, 1.3 | 0.81 |
| Distance to bush forest | Point | 1.17 | 0.88, 1.55 | 0.28 |
|  | 50m | 1.07 | 0.76, 1.52 | 0.70 |
|  | 100m | 1.06 | 0.75, 1.49 | 0.74 |
|  | 150m | 0.98 | 0.7, 1.38 | 0.91 |
|  | 200m | 1.03 | 0.73, 1.44 | 0.87 |
|  | 250m | 1.24 | 0.88, 1.73 | 0.22 |
|  | 300m | 1.22 | 0.88, 1.71 | 0.24 |
|  | 350m | 1.07 | 0.76, 1.5 | 0.71 |
|  | **400m** | **1.29** | **0.92, 1.81** | **0.14** |
|  | 450m | 1.22 | 0.86, 1.73 | 0.27 |
|  | 500m | 1.19 | 0.85, 1.66 | 0.32 |
| Distance to rice agriculture | Point | 0.99 | 0.72, 1.36 | 0.93 |
|  | 50m | 0.81 | 0.58, 1.13 | 0.22 |
|  | 100m | 0.82 | 0.58, 1.13 | 0.23 |
|  | **150m** | **0.79** | **0.57, 1.1** | **0.16** |
|  | **200m** | **0.79** | **0.57, 1.1** | **0.16** |
|  | **250m** | **0.77** | **0.55, 1.08** | **0.13** |
|  | **300m** | **0.80** | **0.57, 1.16** | **0.19** |
|  | 350m | 0.82 | 0.59, 1.14 | 0.23 |
|  | **400m** | **0.79** | **0.57, 1.1** | **0.17** |
|  | 450m | 0.80 | 0.57, 1.13 | 0.20 |
|  | 500m | 0.80 | 0.57, 1.13 | 0.21 |
| Distance to oil palm plantation | **Point** | **0.78** | **0.57, 1.07** | **0.12** |
|  | 50m | 0.81 | 0.57, 1.13 | 0.21 |
|  | 100m | 0.81 | 0.58, 1.12 | 0.20 |
|  | 150m | 0.87 | 0.62, 1.21 | 0.40 |
|  | **200m** | **0.77** | **0.55, 1.07** | **0.12** |
|  | 250m | 0.81 | 0.58, 1.14 | 0.22 |
|  | 300m | 0.89 | 0.64, 1.26 | 0.52 |
|  | 350m | 1.01 | 0.73, 1.41 | 0.94 |
|  | 400m | 1.01 | 0.72, 1.41 | 0.97 |
|  | 450m | 1.06 | 0.8, 1.41 | 0.68 |
| Distance to coconut/ mixed agriculture | Point | 0.98 | 0.71, 1.34 | 0.89 |
|  | 50m | 0.93 | 0.67, 1.28 | 0.65 |
|  | 100m | 0.87 | 0.63, 1.20 | 0.40 |
|  | 150m | 0.85 | 0.61, 1.16 | 0.31 |
|  | 200m | 0.86 | 0.62, 0.18 | 0.35 |
|  | 250m | 0.84 | 0.6, 1.17 | 0.30 |
|  | 300m | 0.93 | 0.69, 1.26 | 0.64 |
|  | 350m | 1.16 | 0.9, 1.5 | 0.27 |
|  | 400m | 1.00 | 0.75, 1.32 | 0.98 |
|  | 450m | 1.11 | 0.87, 1.42 | 0.40 |
| Distance to rubber plantation | **Point** | **1.28** | **0.96, 1.71** | **0.09** |
|  | **50m** | **1.32** | **0.95, 1.82** | **0.10** |
|  | **100m** | **1.49** | **1.1, 2.02** | **0.01** |
|  | 150m | 1.20 | 0.89, 1.62 | 0.23 |
|  | **200m** | **1.27** | **0.93, 1.73** | **0.14** |
| Perimeter: area ratio | 50m | 0.84 | 0.63, 1.12 | 0.23 |
|  | 100m | 0.96 | 0.71, 1.29 | 0.79 |
|  | 150m | 1.23 | 0.89, 1.7 | 0.20 |
|  | **200m** | **1.43** | **1.05, 1.95** | **0.02** |
|  | 250m | 1.19 | 0.88, 1.63 | 0.26 |
|  | **300m** | **1.44** | **1.04, 2** | **0.03** |
|  | 350m | 1.21 | 0.89, 1.67 | 0.23 |
|  | 400m | 1.12 | 0.83, 1.53 | 0.46 |
|  | 450m | 0.91 | 0.66, 1.25 | 0.56 |
|  | 500m | 1.18 | 0.84, 1.67 | 0.34 |
| Shape index | **50m** | **0.82** | **0.61, 1.11** | **0.19** |
|  | 100m | 0.88 | 0.63, 1.24 | 0.47 |
|  | 150m | 1.04 | 0.76, 1.41 | 0.82 |
|  | 200m | 1.16 | 0.83, 1.63 | 0.38 |
|  | 250m | 0.92 | 0.64, 1.32 | 0.65 |
|  | **300m** | **0.77** | **0.52, 1.13** | **0.19** |
|  | 350m | 0.90 | 0.63, 1.27 | 0.56 |
|  | 400m | 0.90 | 0.63, 1.28 | 0.56 |
|  | 450m | 1.25 | 0.89, 1.76 | 0.20 |
|  | 500m | 1.00 | 0.69, 1.44 | 0.99 |
| Fractal dimension | 50m | 0.82 | 0.61, 1.1 | 0.20 |
|  | 100m | 0.90 | 0.65, 1.25 | 0.53 |
|  | 150m | 1.05 | 0.77, 1.42 | 0.77 |
|  | 200m | 1.16 | 0.83, 1.62 | 0.39 |
|  | 250m | 0.92 | 0.65, 1.32 | 0.66 |
|  | **300m** | **0.77** | **0.53, 1.12** | **0.18** |
|  | 350m | 0.88 | 0.63, 1.23 | 0.46 |
|  | 400m | 0.89 | 0.63, 1.26 | 0.50 |
|  | **450m** | **1.32** | **0.94, 1.85** | **0.11** |
|  | 500m | 0.99 | 0.69, 1.41 | 0.95 |
| Density | **Dense** | **0.35** | **0.17, 0.74** | **0.01** |
|  | Planted | 0.96 | 0.37, 1.65 | 0.92 |
|  | Patchy | 0.78 | 0.47, 1.99 | 0.52 |
|  | Sparse | 0.56 | 0.21, 1.5 | 0.25 |
| Diversity | Monoculture | 1.01 | 0.37, 2.77 | 0.99 |
|  | Mixed forest | 1.31 | 0.46, 3.37 | 0.62 |
|  | Farmed-mixed | 1.17 | 0.41, 3.72 | 0.77 |
|  | Edge | 0.73 | 0.26, 2.05 | 0.55 |
|  | **Shrub** | **0.34** | **0.13, 0.91** | **0.03** |
| *Culex* presence | **Point** | **1.39** | **1.1, 1.79** | **0.01** |
| Aedes presence | **Point** | **0.78** | **0.57, 1.07** | **0.12** |

* Variables scaled and mean-centred. Odds ratios increase per SD

Supplementary Information Appendix Table 2: Univariate logistic regression results for water body presence in sampling blocks.

| Variable | OR | 95% CI | P-value |
| --- | --- | --- | --- |
| Aspect | 0.81 | 0.3, 2.17 | 0.67 |
| Slope | 0.04 | 0.006, 0.38 | **0.00** |
| TWI | 102 | 5.76, 1818.68 | **0.00** |
| Distance from large water body | 0.8 | 0.28, 2.29 | 0.682 |
| Elevation | 0.12 | 0.03, 0.44 | **0.00** |
| Rainfall | 2.08 | 0.97, 4.5 | **0.06** |
| Rainfall lagged 1 month | 3.49 | 1.69, 7.22 | **0.00** |
| Rainfall lagged 2 months | 6.22 | 2.37, 16.28 | **0.00** |
| EVI | 1.92 | 0.94, 3.91 | **0.07** |
| NDVI | 0.99 | 0.38, 2.57 | 0.986 |
| Majority of sampling block bush forest |  |  | Ref |
| Majority of block rubber plantation | 0.1 | 0.00, 6.06 | 0.32 |
| Majority of block coconut/mixed plantation | 0.18 | 0.00, 13.67 | 0.28 |
| Majority of block palm oil | 0.27 | 0.00, 30.99 | 0.44 |
| Majority of block rice | 0.35 | 0.01, 42.11 | 0.59 |
| Majority of block built | 0.09 | 0.00, 5.03 | 0.67 |
| Majority of block grassland/ cleared | 0.09 | 0.00, 4.3 | 0.22 |
| No deforestation event occurred in past 24 months |  |  | REF |
| Deforestation event occurred in past 24 months | 0.62 | 0.41 – 0.93 | **0.02** |

Supplementary Information Table 3: Univariate logistic regression results for Anopheles larval presence in sampling blocks.

| Variable | OR | 95% CI | P-value |
| --- | --- | --- | --- |
| Aspect | 0.78 | 0.55, 1.11 | **0.16** |
| Slope | 0.88 | 0.62, 1.26 | 0.49 |
| TWI | 1.04 | 0.73, 1.49 | 0.83 |
| Elevation | 0.82 | 0.6, 1.15 | 0.2 |
| Rainfall | 1.18 | 0.8, 1.85 | 0.49 |
| Rainfall lagged 1 month | 0.95 | 0.6, 1.5 | 0.82 |
| Rainfall lagged 2 months | 0.86 | 0.56, 1.31 | 0.47 |
| EVI | 0.81 | 0.57, 1.16 | 0.25 |
| NDVI | 0.97 | 0.64, 1.47 | 0.87 |
| Majority of block bush forest |  |  | Ref |
| Majority of block rubber plantation | 1.12 | 0.16, 4.51 | 0.874 |
| Majority of block coconut/mixed plantation | 0.78 | 0.16, 3.72 | 0.742 |
| Majority of block palm oil | 0.71 | 0.11, 4.4 | 0.709 |
| Majority of block rice | 0.82 | 0.22 – 9.88 | 0.99 |
| Majority of block built | 0.95 | 0.24, 3.79 | 0.945 |
| Majority of block grassland/ cleared | 0.65 | 0.17, 2.59 | 0.545 |
| No deforestation event occurred in past 24 months |  |  | REF |
| Deforestation event occurred in past 24 months | 1.07 | 0.65 – 1.76 | **0.97** |

Supplementary Information Appendix Table 4: Environmental and spatial covariates included in risk factor analysis, including their resolution, metric and the remote sensing source they were derived from.

| Parameter | Description | Resolution | Source | Metric |
| --- | --- | --- | --- | --- |
| Rainfall | Remote sensing monthly estimated precipitation levels for the study area | 2.5km | NASA TRMM (Tropical Rainfall Monitoring Mission) | mm/day |
| NDVI | Normalised differential vegetation index | 250m | NASA Terra MODIS | 0-1 |
| EVI | Enhanced vegetation index | 250m | NASA Terra MODIS | 0-1 |
| Elevation | Metres above sea level | 30m | NASA Terra ASTER Global Digital Elevation Model (NASA/METI/AIST/Japan Spacesystems, 2019). | Metres |
| Slope, aspect, topographic wetness index (TWI) | Slope: measure of change in elevation  Aspect: compass direction that a slope faces (degrees)  TWI: quantifies control on hydrological processes (Sørensen, Zinko and Seibert, 2006) | 30m | Calculated from NASA Terra Aster Global Digital Elevation Model  (NASA/METI/AIST/Japan Spacesystems, 2019). | Slope: degrees  Aspect: degrees  TWI: 0-1 |
| Distance to houses | Euclidean distance to nearest house | 5m | Classified land cover map of Sabah, prepared as described by (Fornace *et al.*, 2019) | Metres |
| Distance to recent deforestation | Euclidean distance to 2014 deforestation event | 0.1m | Classified time-series of rasters depicting deforestation event. Derived from drone images of the study site and prepared by Stark *et al.* (2019) | Metres |
| Distance to different land cover classifications | Euclidean distance to nearest site of each land cover type | 30m | Classified land cover map of Sabah, with land covers classified as; bush forest, mangrove, rubber plantation, coconut/mixed agriculture, palm oil, rice farm, built environment, grassland/ clearing, intact forest, acacia forest, water. Prepared by Fornace *et al.* (2019). | Metres |
| Vegetation diversity and density | Series of drone images of the study site.  Used to determine vegetation density and diversity surrounding each collection point | 0.1m | Images were collected using Sensefly Unmanned Aerial Vehicle (UAV) during the data collection period in 2015 and 2016. | Density: 4 level factor; Dense, patchy, planted, sparse  Diversity: Monoculture, mixed-forest, farmed-mixed, edge, shrub |
| Fragmentation indices | Perimeter: area ratio, shape index, fractal dimension | 30m | Derived from land cover classification maps | 0-1 |
| Deforestation event (included in sampling block analysis only) | Binary variable for whether a 2014 deforestation event occurred in the sampling block | 0.1m | Classified time-series of rasters depicting deforestation event. Derived from drone images of the study site and prepared by Stark *et al.* (2019) | 0/1 |


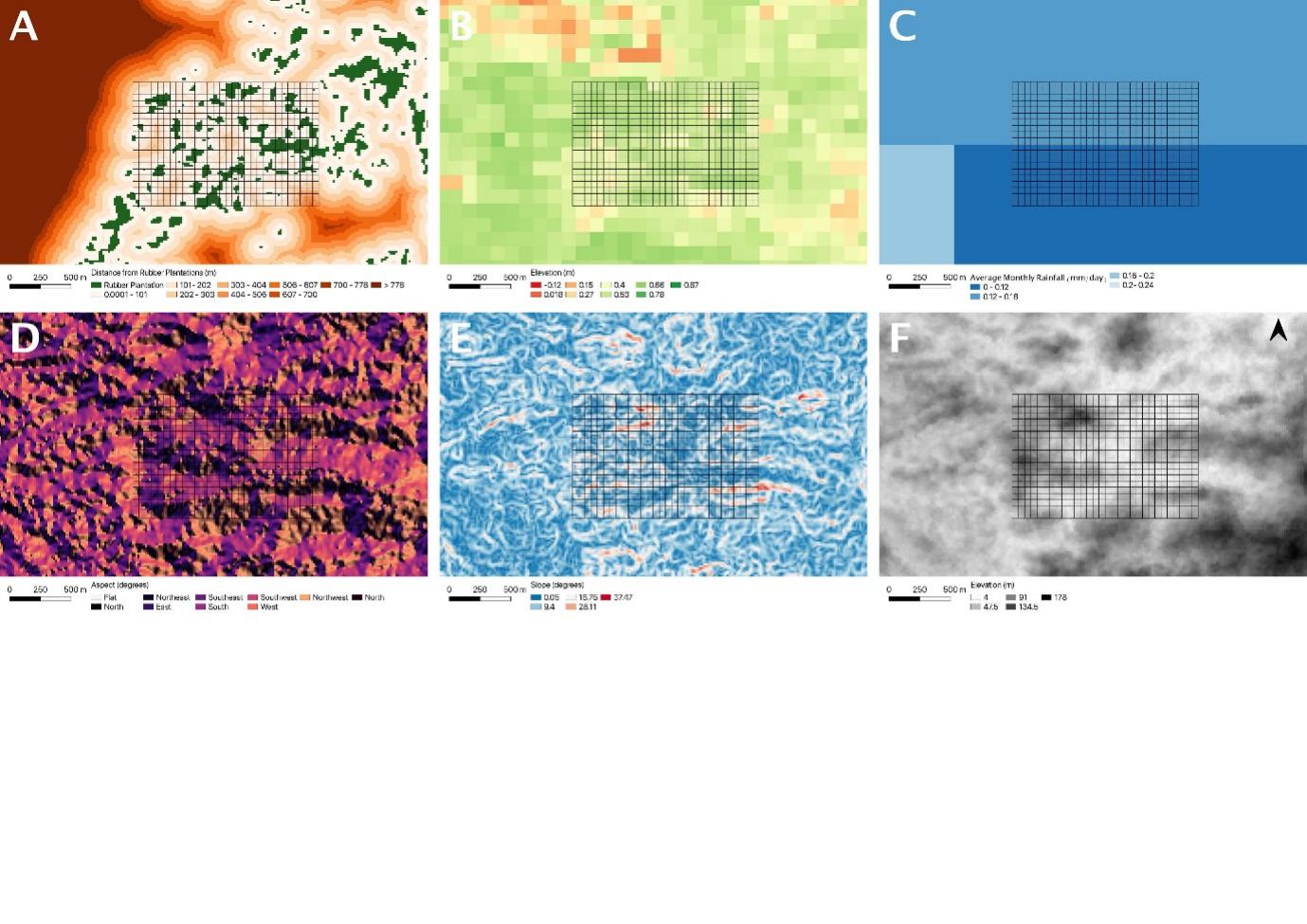


Supplementary Information Figure 1: Examples of satellite derived raster maps and their different spatial resolutions. A) Distance to rubber plantations B) EVI C) Monthly rainfall D) Aspect C) Slope F) Elevation


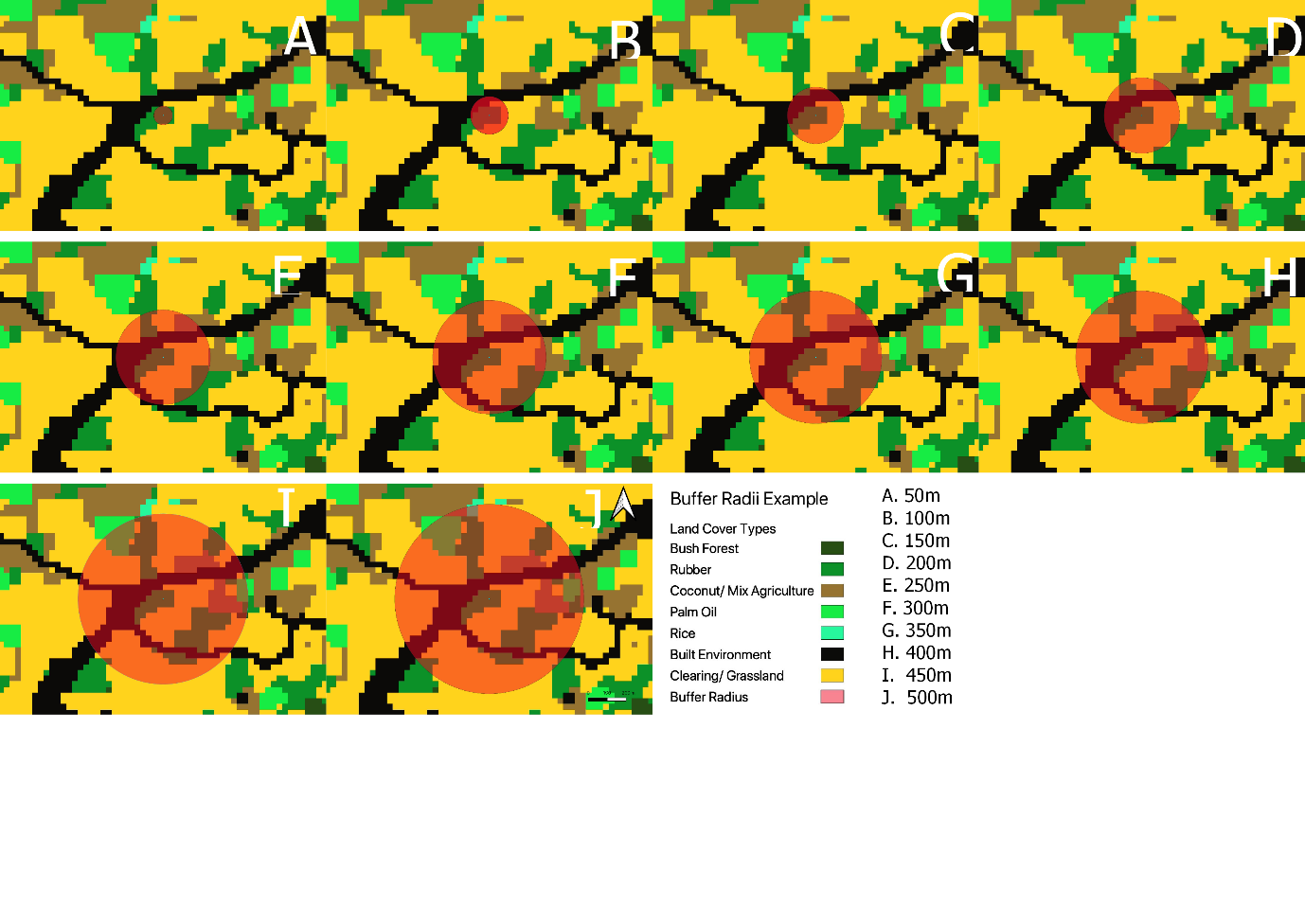


Supplementary Information Figure 2: Example of buffer zones around one water body within the survey site. Mean and standard deviation of each covariate layer (detailed in Table 1) were extracted at the exact water body site and for each buffer radius around each collection point (n=638 *10 buffers)


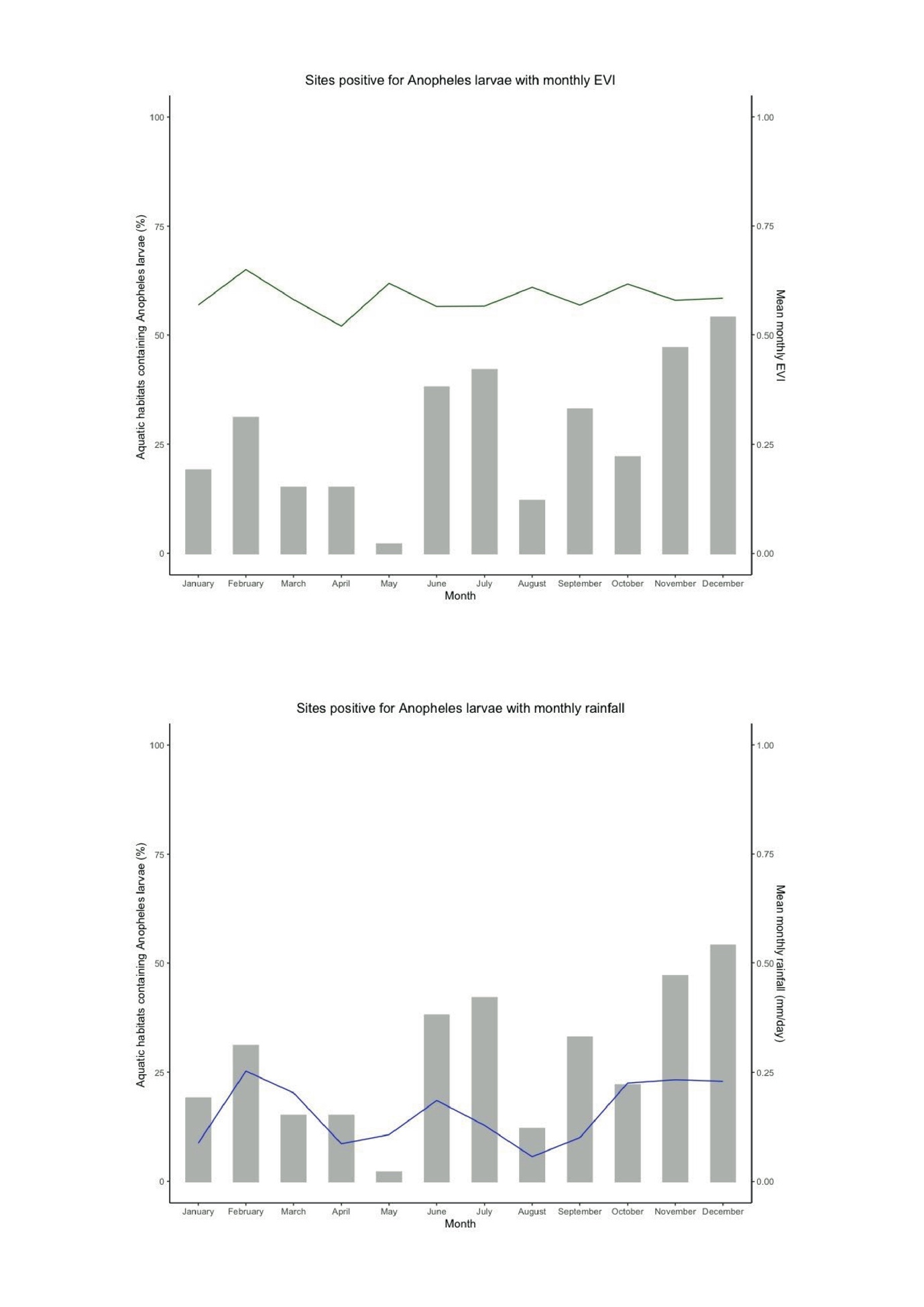


Supplementary Information Figure 3: Percentage of water bodies positive for Anopheles larvae per month with mean monthly rainfall and EVI.
